# Clinical and genetic characteristics of Chinese pediatric and adult patients with hereditary spherocytosis

**DOI:** 10.1186/s13023-024-03290-y

**Published:** 2024-07-24

**Authors:** Chongjun Wu, Yixin Yan, Ting Xiong, Wen Jiang, Jing Xu, Yanfei Rao, Jianyun Ao, Chun Xu, Xuehong Li, Longwang Qi, Wenhong Zheng, Wenjin Li, Zhongjin Xu, Yu Yang, Zhenjiang Li

**Affiliations:** 1 Department of Hematology, Jiangxi Provincial Children’s Hospital, Nanchang, 330000 China; 2 Department of Hematology, The Second Affiliated Hospital, Jiangxi Medical College, Nanchang University, Nanchang, 330000 China; 3 Department of Endocrine Genetics and Metabolism, Jiangxi Provincial Children’s Hospital, Nanchang, 330000 China; 4 Wuhan Third Hospital, Wuhan, 430000 China; 5 Department of Clinical Laboratory, The Second Affiliated Hospital of Nanchang University, Nanchang, 330000 China; 6 Pingxiang People’s Hospital, Pingxiang, 337000 China; 7 The Third People’s Hospital of Jingdezhen, Jingdezhen, 333000 China

**Keywords:** Hereditary spherocytosis, *ANK1*, *SPTB*, Pediatric and adult

## Abstract

**Objective:**

This study aimed to investigate the clinical features, pathogenic gene variants, and potential genotype–phenotype correlations in Chinese patients with hereditary spherocytosis (HS).

**Methods:**

Retrospective analysis of clinical data and molecular genetic characteristics was conducted on patients diagnosed with HS at Jiangxi Provincial Children's Hospital, the Second Affiliated Hospital of Nanchang University, Pingxiang People's Hospital and The Third People's Hospital of Jingdezhen between November 2017 and June 2023. Statistical analyses were performed to compare and analyze the red blood cell (RBC), hemoglobin (HB), mean corpuscular volume (MCV), mean corpuscular hemoglobin (MCH), and mean corpuscular hemoglobin concentration (MCHC) data between and within groups based on different mutations and age groups (< 14 and ≥ 14 years).

**Results:**

A total of 34 HS patients were included in this study, comprising 22 children (64.70%) and 12 adults (35.30%). The probands who underwent genetic testing were derived from 34 unrelated families. Thirty-two variants were tested and 9 of them are novel. Eighteen cases had *ANK1* variants, 15 had *SPTB* variants, and 1 had *SLC4A1* variant. 25 patients performed core family members underwent genetic testing, 17 (68.0%, 17/25) were de novo, 5 (20.0%, 5/25) were maternally inherited, and 3 (12.0%, 3/25) were paternally inherited. *ANK1*-HS patients exhibited more severe anemia compared to cases with *SPTB*-HS, showing lower levels of RBC and HB (*P* < 0.05). Anemia was more severe in patients diagnosed in childhood than in those diagnosed in adulthood. Within the *ANK1*-HS group, MCH levels in adult patients was significantly higher than those in children (*P* < 0.05), while there were no significant differences in RBC, HB, MCV, and MCHC levels between two groups. Adult patients with *SPTB*-HS had significantly higher levels of RBC, HB, and MCH than pediatric patients (*P* < 0.05), while MCV and MCHC levels showed no significant statistical differences.

**Conclusion:**

This study conducted a comparative analysis of phenotypic characteristics and molecular genetics in adult and pediatric patients diagnosed with HS, confirming that pediatric *ANK1*-HS patients exhibit a more severe anemic phenotype compared to *SPTB*-HS patients, while the severity of HS in adults does not significantly differ between different causative genes.

**Supplementary Information:**

The online version contains supplementary material available at 10.1186/s13023-024-03290-y.

## Introduction

Hereditary spherocytosis (HS) is a genetic hematological disorder characterized by various extravascular hemolytic symptoms such as anemia, jaundice, and splenomegaly. It has been reported worldwide, but epidemiological data on HS are limited in China [1]. HS could be diagnosed at any age, and the severity of the phenotype varies among the patients. Wang et al. [2] speculated that the overall prevalence of HS is approximately 1.37/100,000 in Chinese population, making it the most common rare genetic red blood cell membrane defect in China [3]. Currently, most reports on HS in China are based on individual cases or small sample sizes. Some cases present with atypical clinical features, lack a clear family history, and have no definitive laboratory diagnostic markers, making diagnosis and differential diagnosis challenging. HS exhibits genetic and phenotypic heterogeneity, resulting in misdiagnosis or delayed diagnosis until adulthood for some patients [4–6].

The pathogenesis of HS is attributed to genetic variants in red blood cell membrane protein genes, including *ANK1*, *SPTB*, *SPTA1*, *SLC4A1*, and *EPB42*, which lead to HS subtypes 1 to 5. The pathogenic genes and hot spots of variation that cause HS vary widely in different regions, which is related to different races and genetic backgrounds [7], and may also be related to the differences in prevalence between different ethnic regions.

With the increasing popularity of genetic testing techniques such as whole-exome sequencing (WES), more HS patients can be diagnosed based on genetic evidence. In our previous study [8], informations on 15 patients has been reported (ID 1-14 and 31, in this study). We found that *ANK1* and *SPTB* variants are predominant among Chinese HS patients, with nonsense and frameshift mutations being the most common types. However, there were discrepancies between our findings and data from other research centers in China. Genotype–phenotype correlation analysis has seldom been investigated in HS research, and there is controversy regarding the association between different genotypes and phenotypes. Furthermore, the relationship between mutations in different structural domain sites and clinical phenotypes remains debated. Additionally, there is limited research on the clinical and molecular biological differences in HS phenotypes between pediatric and adult patients.

In this study, we summarized and analyzed the clinical and genetic characteristics of 34 HS patients, investigating the genetic and clinical correlations based on the causative genes or structural domains. We also compared the molecular genetics and clinical phenotypes of HS patients aged < 14 (pediatric) and ≥ 14 years (adults).

## Materials and methods

### Study subjects

Informed consent forms were signed by the patients themselves or their guardians for all participants included in the study. Clinical data were collected from 34 HS patients admitted to Jiangxi Provincial Children's Hospital, the Second Affiliated Hospital of Nanchang University, Pingxiang People's Hospital and The Third People's Hospital of Jingdezhen, from November 2017 to June 2023, including both inpatient and outpatient cases. The diagnosis of HS was confirmed in all 34 probands through genetic testing following clinical assessment. And all patients were excluded from other common hereditary hemolysis, include thalassemia. The clinical data of the included cases were obtained from pre-splenectomy test results.

### Genetic testing

After clinical diagnosis, peripheral blood samples were collected from the probands and their family members and sent for genetic tests (by the Beijing Chigene Translational Medicine Research Center Ltd. and the Kaiang Medical Genetic Testing Company). Whole-exome sequencing (WES) was performed to test causative variants and Sanger sequencing was performed to confirm the candidate variants. The human hg19 reference genome sequence was used for mapping sequencing data. Data from the 1000 Genomes (https://www.internationalgenome.org/), ClinVar (https://www.ncbi.nlm.nih.gov/clinvar/), dbSNP (https://www.ncbi.nlm.nih.gov/snp/), gnomAD (https://gnomad.broadinstitute.org/), and LOVD (https://www.lovd.nl/) databases were utilized to annotate the Minor Allele Frequency (MAF) of the candidate variants. The transcript sequences for the *ANK1*, *SPTB*, and *SLC4A1* genes refer to NM_001142446.2, NM_001024858.4, and NM_000342.4, respectively.

### Statistical methods

In this study, a comparative analysis was performed on the clinical data of patients with *ANK1*-HS and *SPTB*-HS. In addition, these patients were divided into two different groups based on their age: paediatric patients under the age of 14 years and adult patients over the age of 14 years. The Mann–Whitney test was used to compare the groups, with statistical significance set at *P* < 0.05.

## Results

### Clinical characteristics of 34 HS patients

A total of 34 patients diagnosed with HS from unrelated families were included in this study. The detailed clinical characteristics of the probands are shown in Table 1. The median age at diagnosis was 9 years and 6 months. Among the 34 patients, there were 13 males (38.23%) and 21 females (61.76%). There were 22 pediatric patients (< 14 years old, 64.70%) and 12 adult patients (≥ 14 years old, 35.30%). All patients had anemia, and in addition, 32 (94.1%) had elevated bilirubin levels, 16 (47.1%) had splenomegaly, and 10 cases (29.4%) had gallstones.

**Table 1:** Clinical characteristics of the probands

Clinical characteristics	Parameter	ANK1	SPTB
Male, n (%)	13 (38.23%)	8 (44.44%)	4 (26.67%)
Pediatric Patients, n (%)	22 (64.70%)	14 (77.78%)	8 (53.33%)
PLT (× 10^9^/L), average (range)	272 ± 136.74 (67–604)	313 ± 158.19 (67–604)	229 ± 77.85 (92–342)
RBC (× 10^12^/L), average (range)	2.46 ± 0.54 (1.51–3.61)	2.21 ± 0.42 (1.51–2.91)	2.81 ± 0.49 (1.79–3.61)
Hb (g/L), average (range)	72.15 ± 17.14 (33–109)	63.78 ± 15.57 (33–93)	82.53 ± 13.79 (61–109)
Ret (%), average (range)	11.10 ± 4.81 (1.75–20.37)	11.07 ± 4.91 (1.75–20.37)	11.14 ± 5.08 (4.05–19.48)
MCV (fL), average (range)	87.59 ± 7.72 (70.2–110.3)	86.99 ± 8.94 (70.2–110.3)	87.07 ± 4.29 (79–93.5)
MCH (pg), average (range)	29.51 ± 2.95 (24.3–38)	29.28 ± 3.16 (24.3–38)	29.22 ± 1.62 (26.8–32.3)
MCHC (g/L), average (range)	331.94 ± 22.68 (297–416)	330.44 ± 26.90 (297–416)	332.47 ± 17.45 (307–358)
HCT (%), average (range)	21.20 ± 4.70 (11–32)	18.78 ± 3.54 (11–32)	24.24 ± 4.46 (15.4–31.8)
T-Bil (μmol/L),average (range)	98.49 ± 99.04 (7.1–436.06)	60.14 ± 40.35 (7.1–147.13)	142.80 ± 131.41 (41–436.06)
D-Bil (μmol/L), average (range)	23.47 ± 41.26 (4.1–212.65)	13.03 ± 4.70 (4.1–28.8)	36.18 ± 60.74 (9.7–212.65)
I-Bil (μmol/L), average (range)	75.01 ± 66.42 (3–290.90)	47.08 ± 39.22 (3–129.83)	106.62 ± 80.32 (26–290.90)
Splenomegaly, n (%)	16 (47.1%)	8 (50%)	8 (50%)
Cholecystolithiasis, n (%)	10 (29.4%)	4 (40%)	6 (60%)

### Spectrum of pathogenic variants and distribution of variant loci in HS patients

**Fig. 1:**
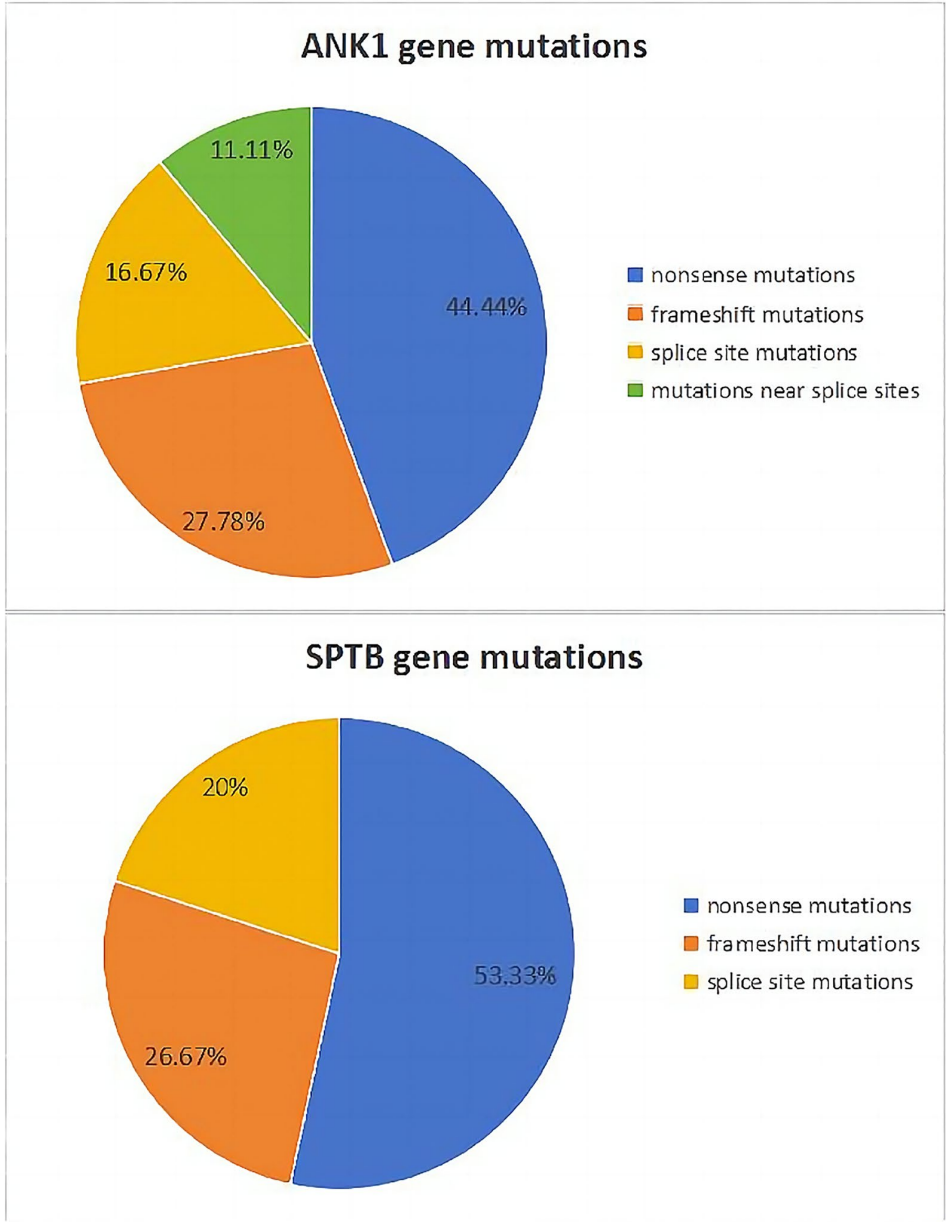
Mutation types of *ANK1* and *SPTB* gene patients

**Fig. 2:**
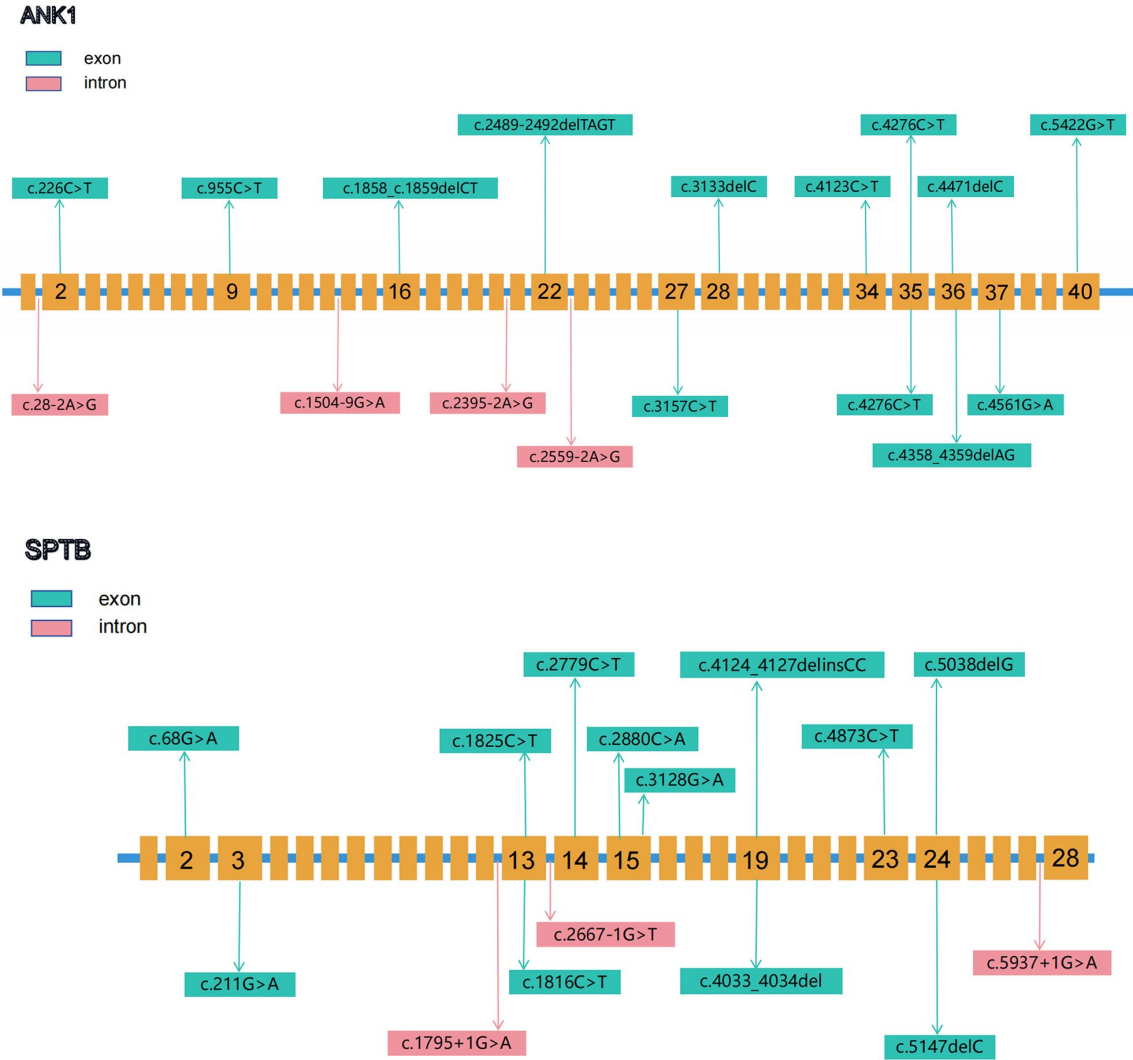
Mutation sites of *ANK1* and *SPTB* genes in HS patients

The genetic variants in the 34 HS patients are shown in Table 2, all of which were compound heterozygous variants. Among the 34 HS genetic variants, 30 were classified as pathogenic according to the ACMG classification, and 4 were classified as likely pathogenic. Among them, nine variants have not been reported before (ID 16, 17, 19, 20, 28, 29, 30, 33 and 34). There were 18 cases (52.94%) with *ANK1* variants, 15 cases (44.12%) with *SPTB* variants, and 1 case (2.94%) with *SLC4A1* variants. No variants were observed in the *SPTA1* and *EPB42* genes. The types of variants included 17 nonsense mutations (50%), 9 frameshift mutations (26.47%), 6 splice site mutations (17.65%), and 2 adjacent to splice sites (5.88%). Among the 18 *ANK1* gene mutations, there were 8 nonsense mutations (44.44%), 5 frameshift mutations (27.78%), 3 splice site mutations (16.67%), and 2 mutations near splice sites (11.11%). Among the 15 *SPTB* mutations, there were 8 nonsense mutations (53.33%), 4 frameshift mutations (26.67%), and 3 splice site mutations (20%) (Fig. 1). The *ANK1* c.4276C > T variant was found in both patients 7 and 12. The *ANK1* c.1504-9G > A variant was found in both patients 15 and 31. Pedigree analysis was performed in 25 families, with 5 cases originating from the maternal side (20.0%), 3 cases from the paternal side (12.0%), and 17 cases showing de novo mutations (68.0%). Among the *ANK1* gene mutations, 13 were in exons and 5 located in introns. Among the *SPTB* gene mutations, 12 were in exons and 3 in introns (Fig. 2).

**Table 2:** Identification of genetic mutations in spherocytosis using genome sequencing

ID	Gene	Chromosomal location	Coding	Protein
1	*SPTB*	chr14:65241215	c.4873C > T	p.R1625*
2	*ANK1*	chr8:41545696-41545697	c.4358_4359del	p.E1453Afs*46
3	*ANK1*	chr8:41559136-41559139	c.2489_2492del	p.L830Sfs*7
4	*ANK1*	chr8:41547849	c.4123C > T	p.R1375*
5	*SPTB*	chr14:65260556	c.1825C > T	p.Q609*
6	*ANK1*	chr8:41559664	c.2395-2A > G	
7	*ANK1*	chr8:41546059	c.4276C > T	p.R1426*
8	*ANK1*	chr8:41615556	c.226C > T	p.Q76*
9	*ANK1*	chr8:41571714-41571715	c.1858_1859del	p.L620Afs*33
10	*SPTB*	chr14:65253555	c.3128G > A	p.W1043*
11	*SPTB*	chr14:65253803	c.2880C > A	p.C960*
12	*ANK1*	chr8:41546059	c.4276C > T	p.R1426*
13	*SPTB*	chr14:65258462	c.2779C > T	p.Q927*
14	*ANK1*	chr8:41580696	c.955C > T	p.R319*
15	*ANK1*	chr8:41573376	c.1504-9G > A	
16	*ANK1*	chr8:41552800	c.3133del	p.R1045Afs*6
17	*SPTB*	chr14:65239969	c.5147del	p.P1716Rfs*10
18	*SPTB*	chr14:65236307	c.5937 + 1G > A	
19	*ANK1*	chr8:41525880	c.5422G > T	p.E1808*
20	*ANK1*	chr8:41542161	c.4561G > A	P.L1521M
21	*SLC4A1*	chr17:42334876	c.1468C > T	p.R490C
22	*ANK1*	chr8:41555641	c.2682-2A > G	
23	*ANK1*	chr8:41615657	c.127-2A > G	
24	*ANK1*	chr8:41552280	c.3280C > T	p.R1094*
25	*SPTB*	chr14:65240078	c.5038del	p.E1680Kfs*32
26	*SPTB*	chr14:65260565	c.1816C > T	p.Q606*
27	*SPTB*	chr14:65261184	c.1795 + 1G > A	
28	*SPTB*	chr14:65258575	c.2667-1G > T	
29	*SPTB*	chr14:65249240_65249241	c.4033_4034del	p.Q1345Vfs*45
30	*SPTB*	chr14:65249148	c.4124_4126delTCTinsC	p.L1375Pfs*15
31	*ANK1*	chr8:41573376	c.1504-9G > A	
32	*SPTB*	chr14:65271746	c.211G > A	p.V71M
33	*SPTB*	chr14:65289745	c.68G > A	p.W23*
34	*ANK1*	chr8:41542128	c.4594del	p.R1532Afs*3

### Genotype–phenotype correlation analysis in HS patients

**Fig. 3:**
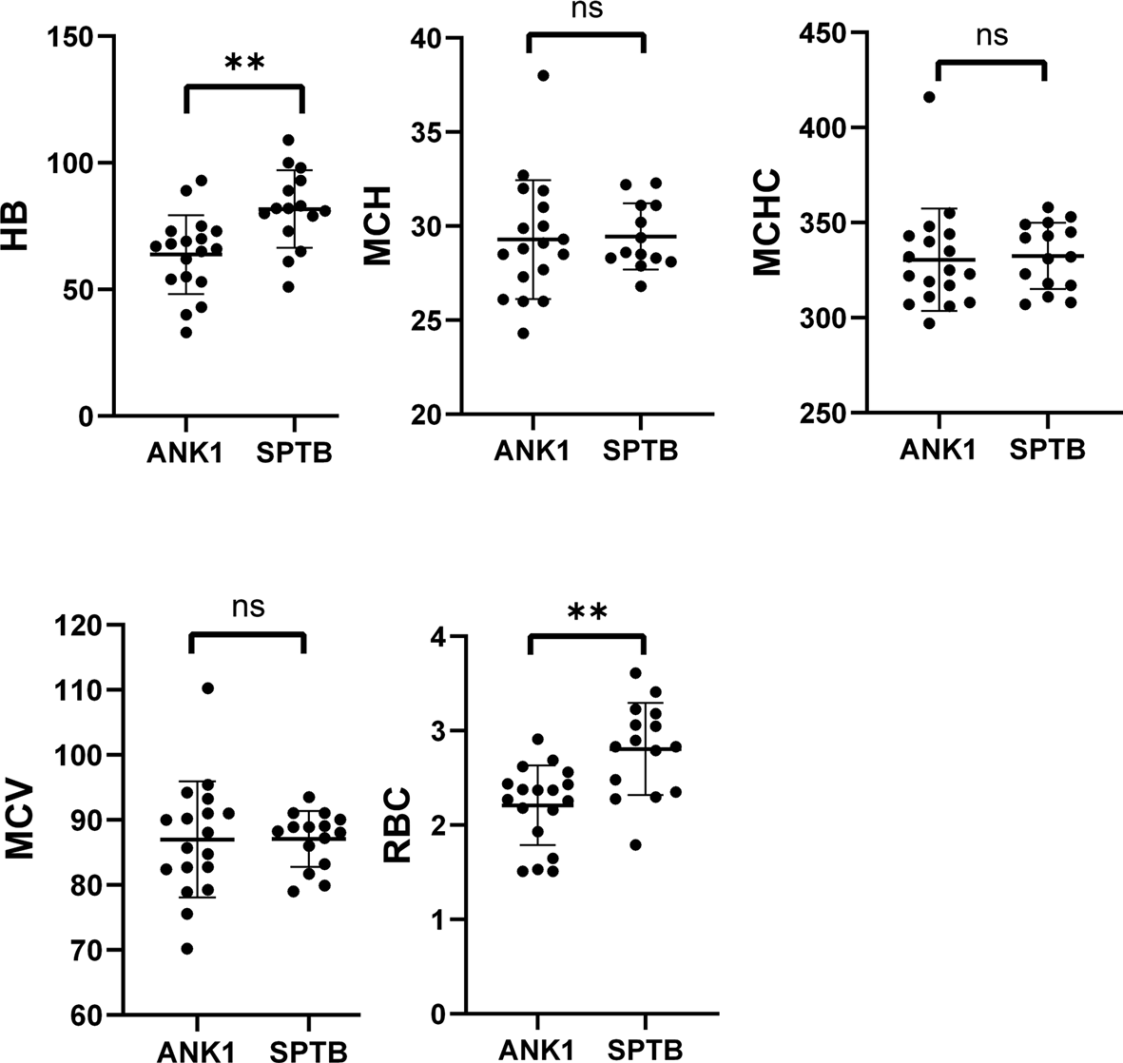
Genotypic-phenotypic correlations in patients with HS

Excluding one case of *SLC4A1*-HS, a comparison of Hb, RBC, MCV, MCH, and MCHC indices between *ANK1*-HS and *SPTB*-HS groups revealed that RBC (*P* = 0.0013) and HB (*P* = 0.0032) were significantly lower in *ANK1*-HS patients compared to *SPTB*-HS patients, while there was no significant difference in MCV, MCH, and MCHC between the two groups. This indicates that *ANK1*-HS patients have more severe anemia phenotype compared to *SPTB*-HS patients (Fig. 3).

### Age-genotype–phenotype correlation in HS patients

**Fig. 4:**
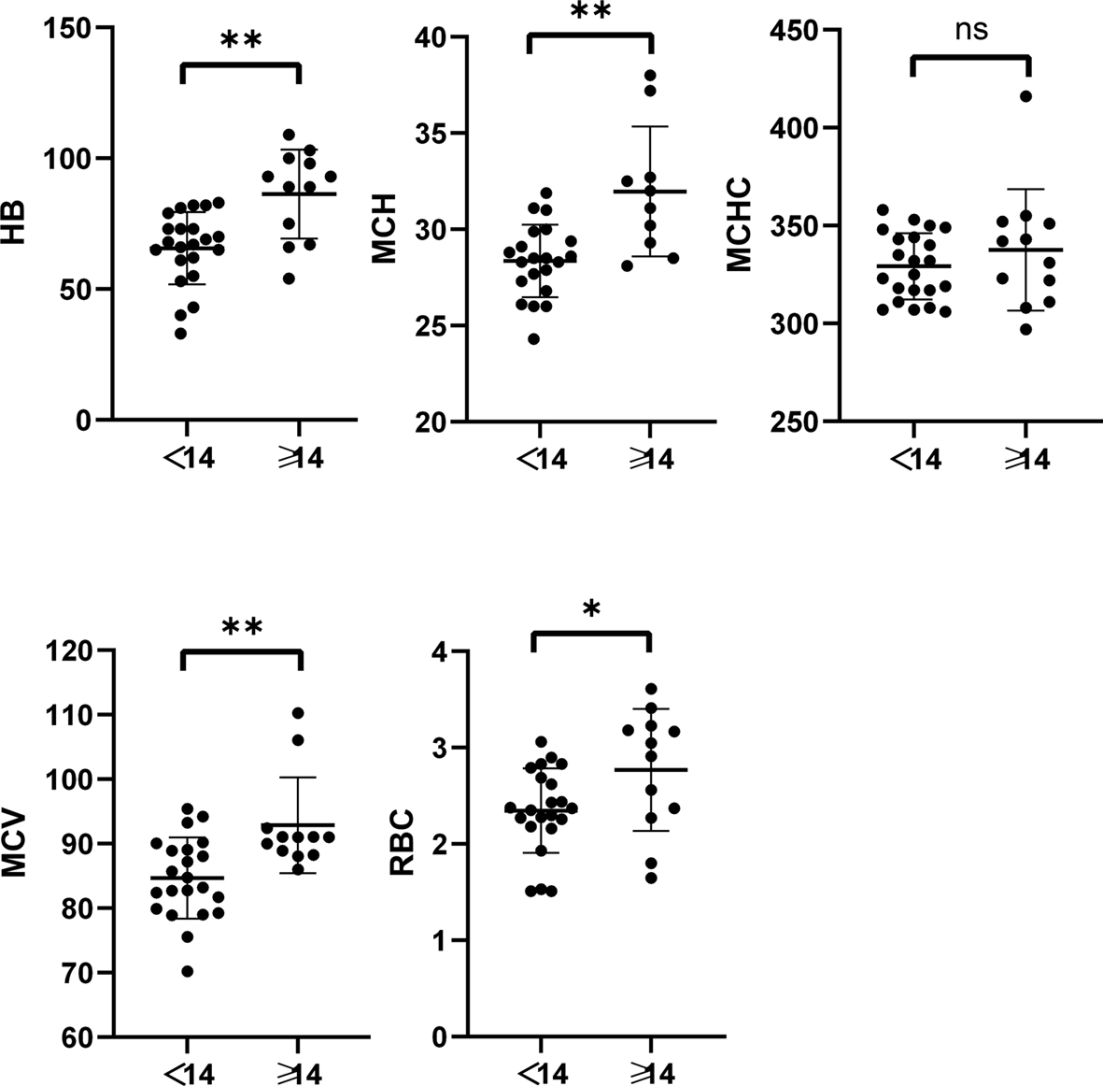
Comparison of Hb, RBC, MCV, MCH, and MCHC levels between adult (12 cases) and pediatric (22 cases) groups

**Fig. 5:**
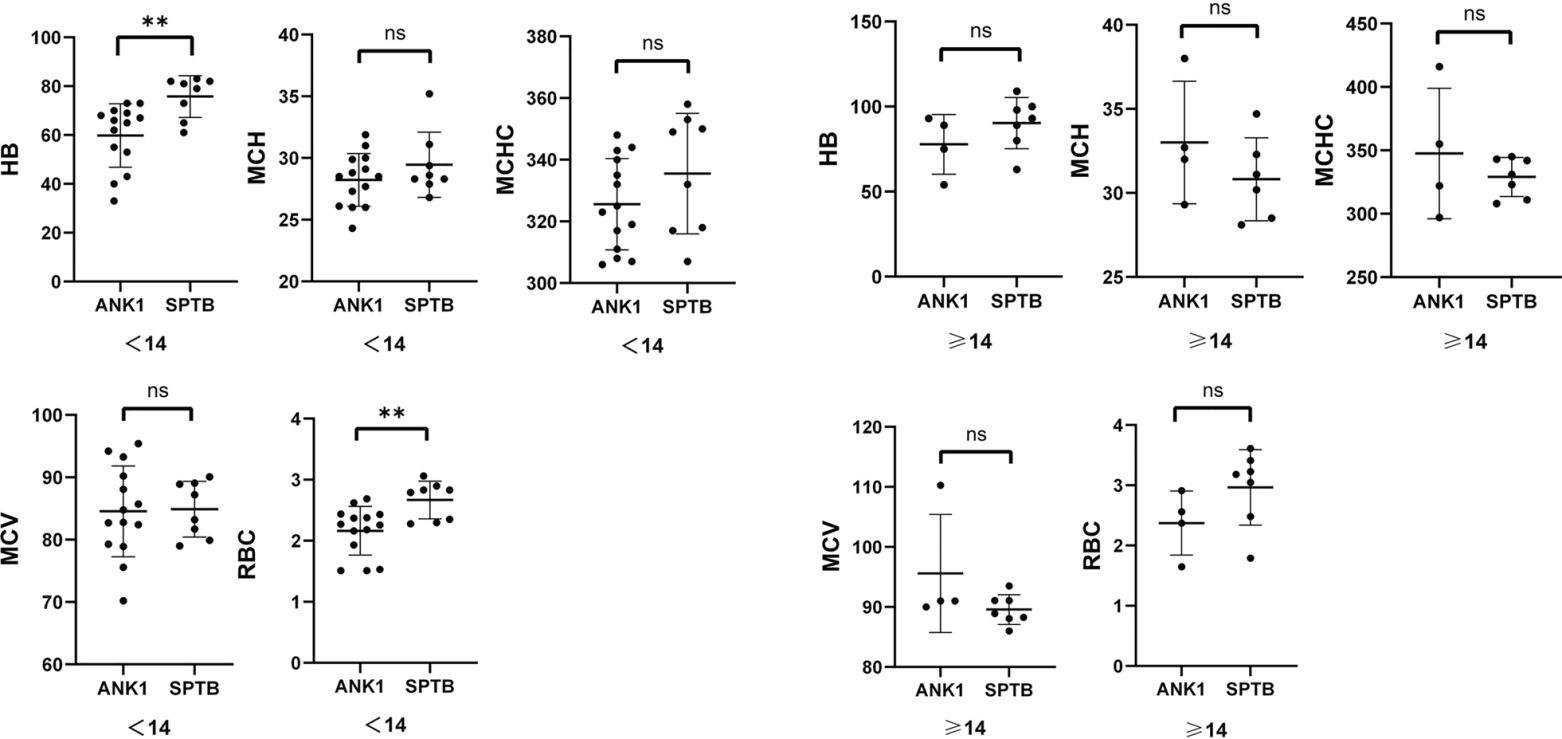
Comparison of Hb and RBC levels between the variant genotypes (*ANK1*, *SPTB*) based on children and adults with HS

**Fig. 6:**
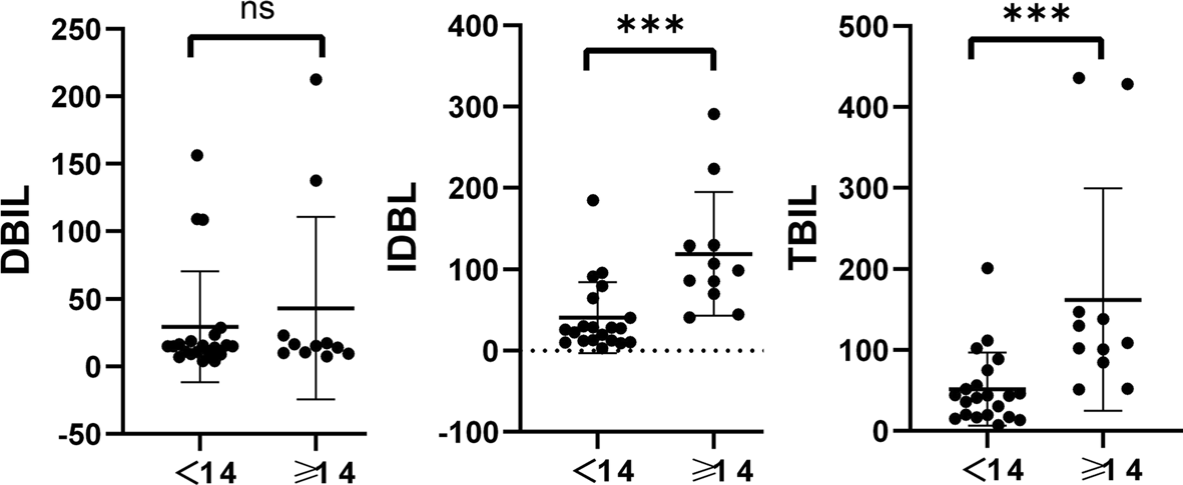
Comparison of TBIL, DBIL, and IDBL levels between adult (12 cases) and pediatric (22 cases) groups

Comparison of Hb, RBC, MCV, MCH, and MCHC levels between adult (12 cases) and pediatric (22 cases) groups revealed that pediatric HS patients had significantly lower Hb (*P* = 0.0015), RBC (*P* = 0.0298), MCV (*P* = 0.0023), and MCH (*P* = 0.0016) compared to adult patients(Fig. 4). Furthermore, when comparing the levels of Hb, RBC, MCV, MCH, and MCHC between *ANK1*-HS and *SPTB*-HS subgroups, it was found that pediatric *ANK1*-HS patients had significantly lower RBC (*P* = 0.012) and Hb (*P* = 0.0104) compared to *SPTB*-HS patients, while there were no significant differences in HB and RBC levels between *ANK1*-HS and *SPTB*-HS patients in the adult group. This suggests that the anemia phenotype is more severe in pediatric *ANK1*-HS patients compared to pediatric *SPTB*-HS patients (Fig. 5). Additionally, TBIL, DBIL, and IDBL levels were significantly lower in pediatric patients compared to the adult group (*P < 0.05, **P < 0.01, ***P < 0.005), indicating a more severe jaundice phenotype in adult HS patients compared to pediatric HS patients (Fig. 6).

**Fig. 7:**
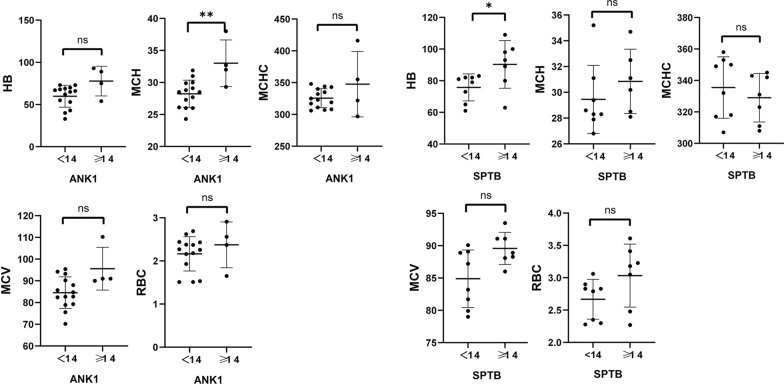
Comparison of Hb and RBC levels between children and adults with HS based on the variant genotypes (*ANK1*, *SPTB*)

**Fig. 8:**
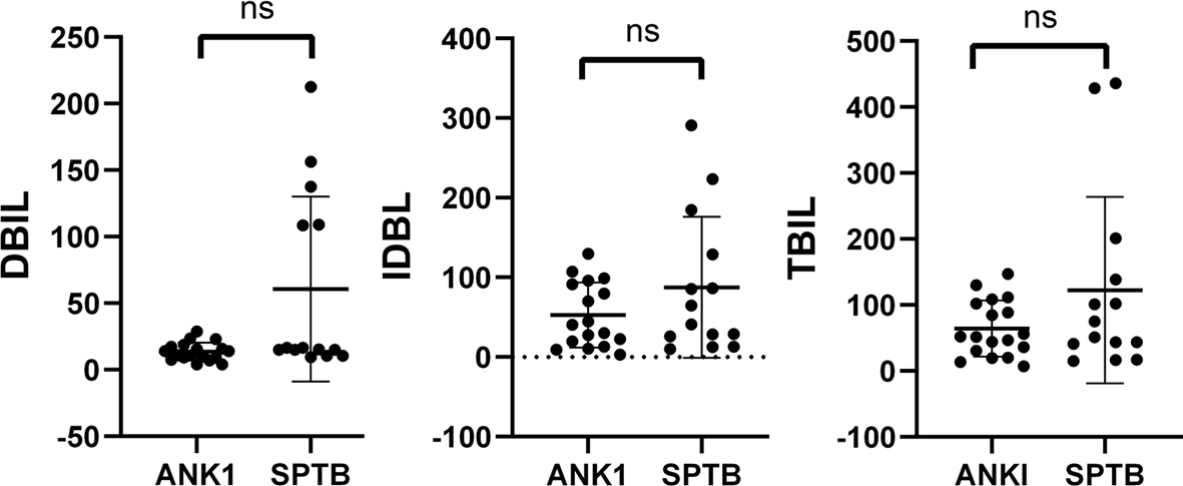
Comparison of TBIL, DBIL, and IDBL levels between *ANK1*-HS and *SPTB*-HS groups

When comparing Hb and RBC levels between children and adults with HS based on the variant genotypes (*ANK1, SPTB*), it was found that Hb levels were significantly lower in pediatric *SPTB*-HS patients compared to adult patients (*P* = 0.0499), while there were no significant differences in other laboratory parameters between the groups (Fig. 7). No significant differences were observed in TBIL, DBIL, and IDBL levels between different genotypes (Fig. 8).

### Genotype–phenotype correlation in HS patients with different mutation types

**Fig. 9:**
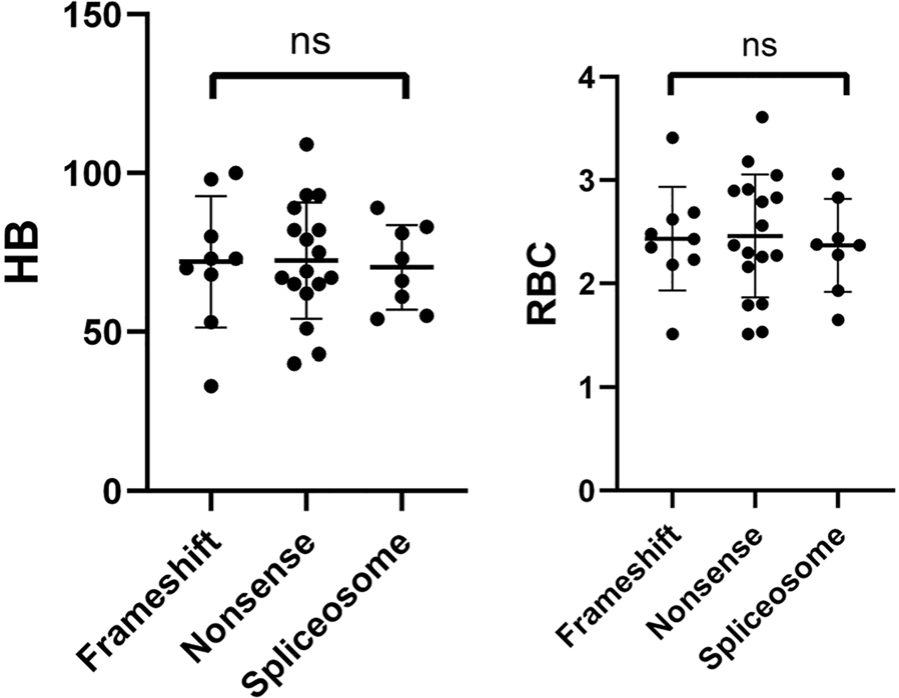
Genotype–phenotype correlation in HS patients with different mutation types

HS patients were grouped based on mutation types, including frameshift mutations (9 cases), nonsense mutations (17 cases), and splice site variants (8 cases). The comparison of Hb and RBC levels among these three groups showed no significant differences in these two parameters (Fig. 9).

### Variability of genotype–phenotype in families of HS patients

**Fig. 10:**
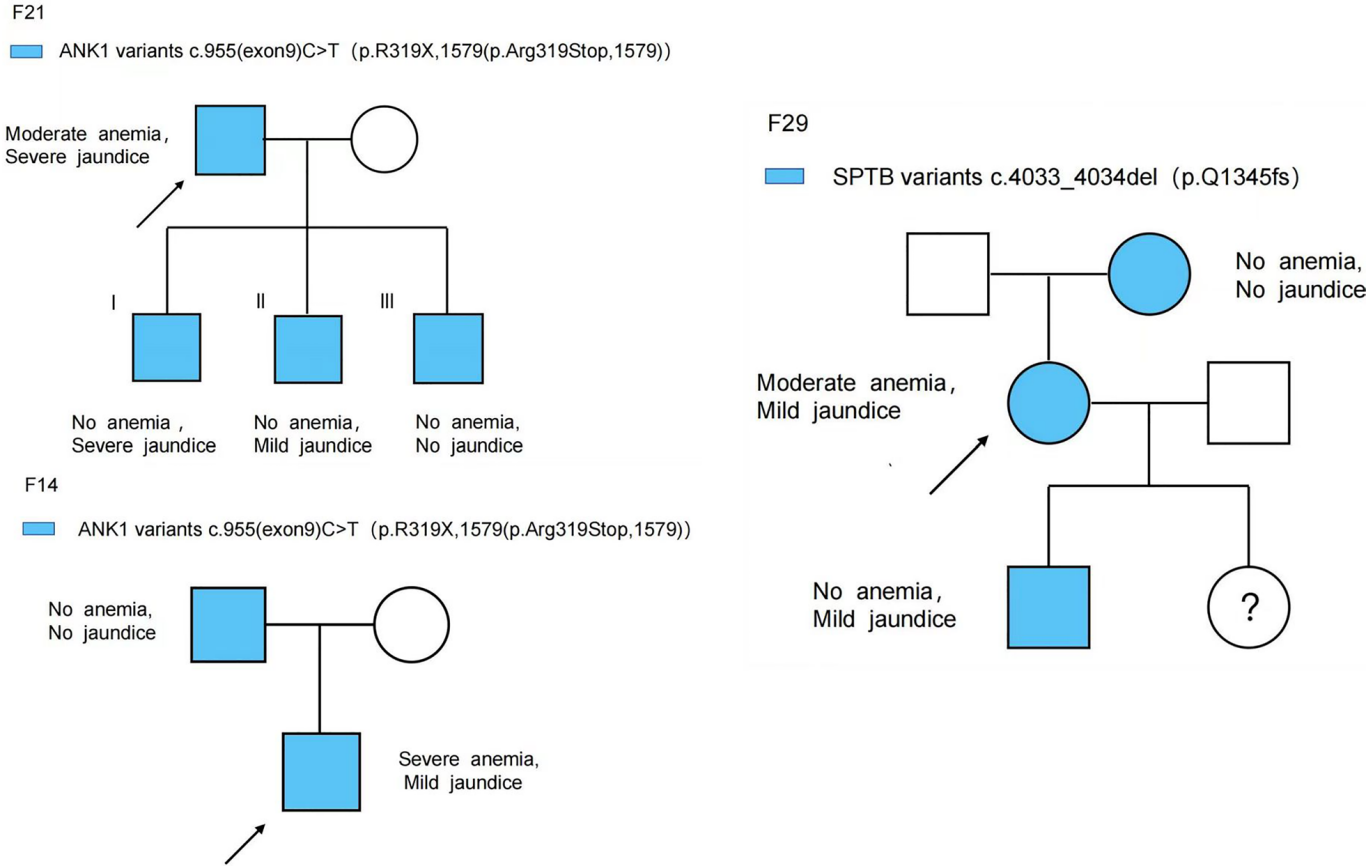
Variability of genotype–phenotype in families of HS patients

To further investigate the relationship between genotype and phenotype, we examined 21 HS patient families out of the total 34 patients and identified three families with phenotypic heterogeneity (Fig. 10).

In Family 21, the father and three children were diagnosed with *SLC4A1*-HS. The father exhibited moderate anemia with mild jaundice, while the three offspring showed no signs of anemia. Offspring I and II presented with severe and mild jaundice, respectively, while Offspring III showed no apparent signs of jaundice.

In Family 14, the index case was diagnosed with *ANK1*-HS, inheriting the *ANK1* gene mutation from the biological father who had no HS phenotype.

In Family 29, the grandmother, mother, and children of three generations shared the same *SPTB* mutation. The mother had moderate anemia and mild jaundice, the children did not have anemia but showed mild jaundice, while the grandmother was an asymptomatic carrier of the gene mutation.

## Discussion

Hereditary spherocytosis (HS) is the most common form of inherited hemolytic anemia caused by abnormalities in red blood cell membrane proteins. The known causative genes of HS include *ANK1* (OMIM: *612641), *SPTB* (OMIM: *182870), *SLC4A1* (OMIM: *109270), *SPTA1* (OMIM: *182860), and *EPB42* (OMIM: *177070) and the majority of them exhibit autosomal dominant inheritance, while a minority demonstrate autosomal recessive inheritance. Clinical manifestations of HS show heterogeneity. Typical HS patients usually present with anemia and jaundice in childhood, and as they grow older, they develop splenomegaly, gallstones, and other complications. Diagnosis was previously made through peripheral blood smear examination and red blood cell fragility tests, but now genetic sequencing can provide a definitive diagnosis by identifying the underlying genetic mechanisms. Although all adult patients in our cohort exhibited typical symptoms such as anemia, jaundice, splenomegaly, and gallstones, two adult patients were initially misdiagnosed with autoimmune hemolytic anemia, which tested negative for direct antiglobulin test (DAT). These patients received treatment with intravenous immunoglobulin, corticosteroids, and cyclosporine, but due to poor response, the diagnosis was reassessed. Reasons for misdiagnosis of HS include the absence of a family history, the presence of spherocytes in autoimmune hemolytic anemia, a relatively low percentage of spherocytes in the peripheral blood, and the misconception that jaundice, splenomegaly, and gallstones are not specific to HS. Limited awareness of the disease among physicians also contributes to misdiagnosis. We recommend that genetic testing for children and adults with unexplained hemolytic anemia to establish a definitive diagnosis at the genetic level.

The incidence and spectrum of pathogenic gene mutations vary among different races and regions. Wang et al. [9]summarized the data of Chinese HS patients from 2000 to 2020, and the proportions of *ANK1*, *SPTB*, *SLC4A1*, and *SPTA1* mutations were 46%, 42%, 11%, and 1%, respectively, which is similar to the research data from Korea [10]. Our study confirmed previous findings: *ANK1* mutations accounted for 52.94%, *SPTB* mutations accounted for 44.12%, *SLC4A1* mutations accounted for 2.94%, and no *SPTA1* or *EPB42* mutations were found. In addition, *ANK1* variants account for 40–65% in the United States and Europe, 5–10% in Japan [11], and 27% in the Netherlands [12], suggesting that the pathogenicity rate of *ANK1*-HS in China is closer to that of Europe and the United States, while other East Asian regions, such as Japan, may be limited by sample size in research.

Through pedigree analysis, it was found that 32.0% (8/25) of mutations were inherited within families, while 68.0% (17/25) were de novo mutations. Additionally, In our current study, 9 mutations were previously unreported. Miraglia et al. [13] analyzed core pedigrees of 19 HS patients, of which 12 cases (63%) were de novo mutations. Peng et al. [14] conducted pedigree analysis of 16 HS cases, with 6 cases (37.5%) being inherited mutations and 10 (62.5%) being de novo. This indicates the importance of proactive and early molecular diagnosis for HS, as it has significant implications for genetic counseling. However, in early diagnosis, physicians may miss the opportunity for a definitive diagnosis due to a lack of genetic evidence and milder phenotypes. Furthermore, the incomplete penetrance observed in 3 families (Family 14, 29, and 21) in our study highlights the importance of proactive molecular diagnosis. Van Vuren et al. [12] suggested that phenotypic heterogeneity within affected individuals in a family may be influenced by factors such as functional compensation by normal alleles in the heterozygous state, the impact of modifier genes (such as a-LEPRA and a-LELY) [12, 15–17], and the regulatory role of epigenetic modifications on HS gene expression. Additionally, 20–30% of HS patients exhibit compensatory hemolysis that is characterized by elevated indirect bilirubin and reticulocytes but normal hemoglobin levels. These patients may develop anemia with age and require close monitoring of disease progression [12].

There is limited research on the diagnosis of HS patients in children and adults. In this study, a comparison between pediatric and adult diagnosis revealed that anemia was more severe in pediatric patients compared to adult patients. Among pediatric patients, RBC, HB, MCV, and MCH levels were significantly lower than in adult patients. In adult *ANK1*-HS patients, MCH levels were significantly higher than in pediatric patients, but there were no significant differences in RBC, HB, MCV, and MCHC levels. In *SPTB*-HS patients, adults had significantly higher levels of RBC, HB, and MCH compared to children (*P* < 0.05), while MCV and MCHC levels showed no significant differences. Although infants have slightly lower hemoglobin (HB) levels than adults, their bone marrow has a relatively enhanced compensatory capacity. Among this patient group, 17 out of 22 (77.27%) pediatric cases presented with moderate anemia, while 5 out of 22 (22.73%) exhibited severe anemia, and there were no instances of mild anemia. Among the adult patients, there were 5 cases of mild anemia (41.67%, 5/12), 6 cases of moderate anemia (50.0%, 6/12), and 1 case of severe anemia (8.33%, 1/12). It is clear that anemia presents with greater severity in paediatric patients. However, it should be noted that the sample size in this study is relatively limited, necessitating further confirmation through subsequent investigations. Additionally, there were no significant differences in HB and RBC levels between adult *ANK1*-HS and *SPTB*-HS patients. We found that the severity of HS in adult patients does not differ significantly between different gene mutations, which is consistent with some previous research findings. However, in patients under 14 years of age, *ANK1*-HS had more severe anemia compared to *SPTB*-HS, which may be related to the relatively small number of children in previous studies. Our study suggests significant phenotypic differences between pediatric and adult diagnosis of HS, which need to be further confirmed with larger sample sizes.

Among the 34 cases in this study, nonsense mutations accounted for 50%, frameshift mutations accounted for 26.47%, and splice site mutations accounted for 23.53%. This indicates that HS is mainly caused by nonsense, frameshift, and splice site mutations, which is consistent with most reports [10, 18–20], in which only a few studies have reported missense mutations in HS genes [19, 21]. The mutations in our cohort were scattered throughout the exons and introns of the *ANK1* and *SPTB* genes, and no hotspot mutations or regions were identified, which is consistent with previous reports [22].

The relationship between gene variants and phenotype in HS patients is controversial. Aggarwal et al. [20] and Tole et al. [23] have found that the impact of *ANK1* mutations on red blood cell indices is similar to that of *SPTB* mutations, and mutation type or location cannot predict the severity of the disease. On the other hand, Park et al. [10] found that *SPTA1* mutations are associated with the most severe disease, while *SLC4A1* mutations result in the mildest phenotype. Qin et al. [19] observed that MCV and MCH levels were significantly higher in *ANK1*-HS compared to *SPTB*-HS, and the percentage of spherocytes in the peripheral blood was significantly lower in *ANK1* mutation patients. Additionally, the MCHC levels in the nonsense, frameshift, and splice site mutation groups were significantly higher than in the missense mutation group, and the severity of the disease did not differ significantly between different gene mutations. In our study, an analysis of genotype and phenotype revealed that RBC and HB levels in *ANK1*-HS were significantly lower than in *SPTB*-HS, but there were no significant differences in MCV, MCH, and MCHC levels, which differs from previous reports [18, 19]. Additionally, when classified by mutation type, there were no significant statistical differences in HB and RBC counts between the nonsense, frameshift, and splice site mutation groups (*P* > 0.05), which is consistent with the findings of Wang et al. [9].

The retrospective analysis spanned five years and encompassed both children and adults diagnosed in Jiangxi Province, China. It is worth noting that the limited sample size may not be fully representative of comprehensive data on HS patients in China. Some of the conclusions require further verification by increasing the sample size and extending the follow-up period. Disease progression indicators, such as blood count, splenomegaly, and gallstones, are also being actively monitored. Once enough data has been collected, we will conduct further analysis. This report only focuses on identifying new variants in known HS genes due to technical limitations and does not include other possible mutations. It may not cover all genetic factors related to HS, including potential new genes. In the next step, we will analyze other variables such as family background, age group, different environments, and lifestyle factors.

## Conclusion

This study, based on an expanded sample size of Chinese HS patients, analyzed the genotype–phenotype association of HS. The results confirmed that HS patients with *ANK1* defects diagnosed in childhood experience more severe anemia compared to those with *SPTB* defects. However, the severity of HS in adult patients did not differ significantly between different genotypes. Furthermore, the analysis of age and its correlation with different genotypes and phenotypes revealed that anemia is more severe in pediatric patients compared to adult patients.

### Supplementary Information

Supplementary Material 1.

## Data Availability

All data generated or analysed during this study are included in this published article [and its supplementary information files].
