# Supplementary material for: Clinical and genetic characteristics of Chinese pediatric and adult patients with hereditary spherocytosis
Source: Orphanet J Rare Dis. 2024 Jul 24;19:278. doi: 10.1186/s13023-024-03290-y (PMC11267807; doi:10.1186/s13023-024-03290-y)
Supplement: Supplementary file 1 — Supplementary Material 1. [file 13023_2024_3290_MOESM1_ESM.docx]

| ID | Gender | Age | RBC | HB | PLT | HCT | MCV | MCH | MCHC | RDW-SD | RDW-CV | RET# | RET | TBIL | DBIL | IDBL | Color ultrasound |
| --- | --- | --- | --- | --- | --- | --- | --- | --- | --- | --- | --- | --- | --- | --- | --- | --- | --- |
| 1 | Female | 13y6m | 2.3 | 65 | 248 | 20.5 | 89.1 | 28.3 | 317 | 89.1 | 28.7 | 352.36 | 15.32 | 156.3 | 12.93 | 143.37 | Hepatosplenomegaly and Gallstone |
| 2 | Female | 10y8m | 1.51 | 33 | 201 | 10.6 | 70.2 | 31.9 | 311 | 54.9 | 24.2 | 146.17 | 9.68 | 36 | 23.57 | 12.43 | Hepatosplenomegaly and Cholestasis |
| 3 | Female | 4m | 2.43 | 73 | 545 | 22.9 | 94.2 | 30 | 319 | 62 | 18.6 | 322.46 | 13.27 | 20.1 | 7.13 | 12.97 |  |
| 4 | Female | 4y4m | 2.16 | 67 | 184 | 20.6 | 95.4 | 31 | 325 | 81.8 | 24.5 | 369.58 | 17.11 | 46.5 | 18.85 | 27.65 | Splenomegaly |
| 5 | Female | 4m5d | 2.83 | 79 | 279 | 22.6 | 79.9 | 27.9 | 350 | 64.5 | 23.3 | 248.47 | 8.78 | 43.8 | 15 | 28.8 | Splenomegaly |
| 6 | Male | 3y6m | 2.44 | 73 | 330 | 22 | 90.2 | 29.9 | 332 | 62 | 20.7 | 422.36 | 17.31 | 51.7 | 28.8 | 22.9 |  |
| 7 | Male | 1m6d | 2.26 | 65 | 453 | 18.7 | 82.7 | 28.8 | 348 | 48.2 | 16.3 | 132.88 | 5.88 | 111.8 | 15.6 | 96.2 |  |
| 8 | Male | 4y9m | 2.27 | 62 | 243 | 18 | 79.3 | 27.3 | 344 | 59.8 | 21.8 | 116.22 | 5.12 | 19.9 | 9.3 | 10.6 | Splenomegaly |
| 9 | Female | 9m14d | 2.69 | 70 | 351 | 22.8 | 84.8 | 26 | 307 | 88.7 | 30.6 | 323.876 | 12.04 | 13.5 | 4.2 | 9.3 |  |
| 10 | Male | 9y6m | 2.9 | 82 | 291 | 22.9 | 79 | 28.3 | 358 | 53.5 | 18.7 | 142.97 | 4.93 | 41 | 15 | 26 | Splenomegaly |
| 11 | Female | 10y4m | 2.79 | 82 | 342 | 23.2 | 83.2 | 29.4 | 353 | 65.6 | 22.9 | 543.492 | 19.48 | 201.3 | 16.7 | 184.6 | Splenomegaly and Cholestasis |
| 12 | Male | 2m12d | 1.51 | 43 | 268 | 13.3 | 88.1 | 28.5 | 323 | 87.5 | 29.8 | 202.45 | 13.41 | 88.8 | 9 | 79.8 | Splenomegaly |
| 13 | Male | 14y | 3.61 | 109 | 185 | 31.8 | 88.1 | 30.2 | 343 | 57.2 | 18.2 | 297.464 | 8.24 | 51.4 | 10.5 | 40.9 | Cholestasis |
| 14 | Male | 4y4m | 1.53 | 40 | 164 | 12.6 | 82.4 | 26.1 | 317 | 80.1 | 31 | 218.79 | 14.3 | 102.2 | 10.9 | 91.3 | Splenomegaly |
| 15 | Female | 9m30d | 2.38 | 66 | 529 | 19.7 | 82.8 | 27.7 | 335 | 46.1 | 16.5 | 170.64 | 7.11 | 56.3 | 15.8 | 40.5 |  |
| 16 | Female | 10m | 2.62 | 68 | 344 | 19.8 | 75.6 | 26 | 343 | 52 | 19.1 | 45.85 | 1.75 | 7.1 | 4.1 | 3 |  |
| 17 | Female | 12y8m | 2.35 | 73 | 271 | 20.9 | 88.9 | 31.1 | 349 | 72.6 | 23.5 | 399.265 | 16.99 | 109 | 10 | 99 |  |
| 18 | Female | 2m5d | 2.83 | 81 | 304 | 25.5 | 90.1 | 28.6 | 318 | 57.1 | 17.3 | 114.62 | 4.05 | 43.6 | 15.19 | 28.41 |  |
| 19 | Female | 1m24d | 2.37 | 69 | 481 | 20.3 | 85.7 | 29.1 | 340 | 54.3 | 17.8 | 209.03 | 8.82 | 30.8 | 11.13 | 19.67 |  |
| 20 | Female | 23y | 2.56 | 75 | 67 | 23.3 | 91 | 29.3 | 322 |  |  |  | 8.4 | 108.9 | 10.1 | 98.8 | Splenomegaly and Cholestasis |
| 21 | Male | 59y | 1.8 | 67 | 107 | 19.1 | 106.1 | 37.2 | 351 | 72.8 | 18.9 | 211.1 | 11.29 | 130.4 | 23 | 107.4 |  |
| 22 | Female | 51y | 1.65 | 54 | 86 | 18.2 | 110.3 | 32.7 | 297 | 84.2 | 21.6 | 199.2 | 12.07 | 84.94 | 14.15 | 70.35 | Megalosplenia and Cholestasis |
| 23 | Female | 42y | 2.37 | 89 | 604 | 20 | 91 | 38 | 416 |  |  |  | 7.4 | 52.3 | 7.5 | 44.8 |  |
| 24 | Male | 23y | 2.91 | 93 | 187 | 20 | 90 | 32 | 355 | 77.6 | 25.1 | 592.8 | 20.37 | 147.1 | 17.3 | 129.83 | Megalosplenia and Cholestasis |
| 25 | Female | 20y | 3.41 | 98 | 211 | 30.3 | 88.9 |  | 323 |  |  |  | 7.43 | 428.6 | 137.7 | 290.9 |  |
| 26 | Female | 16y | 3.05 | 93 | - | 26 | 88.3 |  | 311 |  |  |  | 8.34 | 138.7 | 9.7 | 129 |  |
| 27 | Male | 8y | 3.06 | 83 | - | 23 | 81.7 |  | 332 |  |  |  | 11.3 | 75.1 | 10.5 | 64.6 |  |
| 28 | Female | 13y | 2.28 | 61 | 186 | 19.9 | 87.2 | 26.8 | 307 | 88.7 | 29.6 | 395 | 17.35 | 108.6 | 12.69 | 95.93 | Splenomegaly and Cholestasis |
| 29 | Female | 44y | 2.48 | 80 | 178 | 23.2 | 93.5 | 32.3 | 345 | 66.5 | 20 | 284.5 | 11.47 | 63.59 | 11.39 | 52.2 | Splenomegaly and Cholestasis |
| 30 | Female | 17y | 3.23 | 100 | 288 | 29.4 | 91.1 | 31.1 | 342 | 63 | 19.1 |  |  |  |  |  |  |
| 31 | Male | 4m18d | 1.93 | 55 | 335 | 18 | 93.3 | 28.5 | 306 | 83.3 | 28.9 | 272.2 | 14.13 | 44.4 | 14.1 | 30.3 |  |
| 32 | Male | 34y | 3.18 | 89 | 92 | 29 | 91.1 | 28.1 | 308 | 90.1 | 27 |  |  | 436.1 | 212.6 | 223.41 | Megalosplenia and Cholestasis |
| 33 | Female | 51y | 2.27 | 63 | 99 | 15.4 | 85.5 | 27.8 | 325 | 65.6 | 21.1 | 82.7 |  | 102.1 | 16.59 | 85.54 | Splenomegaly |
| 34 | Male | 4y4m | 2.18 | 53 | 258 | 17.2 | 78.9 | 24.3 | 308 |  |  |  |  |  |  |  |  |

**Supplementary material** Clinical characteristics of the probands.

Note : WBC : White blood cells(×10^9^ / L ), reference range : 3.85 - 10 ; RBC : red blood cells (×10^12^ / L ), reference range : 3.1 - 4.5 ; HB : Hemoglobin ( g / L ), reference range : 110-149 ; PLT : platelet (×10^9^ / L ), reference range : 100-320 ; HCT : hematocrit ( % ), reference range : 35-45 ; MCV : mean corpuscular volume ( fl ), reference range : 80-98 ; MCH : mean hemoglobin ( pg ), reference range : 25-35 ; MCHC : mean hemoglobin concentration ( g /L ), reference range : 300-360 ; RDW-SD : red blood cell distribution width ( fl ), reference range : 32.3-42.4 ; RDW-CV ( % ) ; RET # : absolute value of reticulocyte (×10^9^ / L ), reference range : 25-75 ; RET : reticulocyte percentage ( % ) ; TBIL : total bilirubin (μmol / L ), reference range : 2-22 ; DBIL : Direct bilirubin (μmol / L ), reference range : 1-8 ; IDBL : Indirect bilirubin (μmol / L ), reference range : 1-19 ; y: year, m: month.
